# Necrotizing fasciitis following gastrostomy tube placement, detected by point-of-care ultrasound, case report

**DOI:** 10.1016/j.ijscr.2023.108889

**Published:** 2023-10-01

**Authors:** Zouheir Ibrahim Bitar, Ossama Sajeh Maadarani, Mahmoud Mostafa Elzoueiry, Aishah Alfarhan, Mohamed Elsayed Elhabibi

**Affiliations:** a Critical Care Unit, Ahmadi Hospital, Kuwait Oil Company, POBOx 46468, Postal code 64015 Fahahil, Kuwait; b Critical Care Unit, Internal Medicine Dept, Ahmadi Hospital, Kuwait Oil Company, Kuwait; c Critical Care Unit, Internal Medicine Dept, Ahmadi Hospital, Kuwait; d Royal College Of Surgeons in Ireland (RCSI), Kuwait; e Critical Care Unit, Ahmadi Hospital, Kuwait Oil Company, Ahmadi, Kuwait

**Keywords:** Case report, Deep fasciitis, POCUS

## Abstract

**Introduction:**

Necrotizing fasciitis is a recognized rare complication of gastrostomy tube replacement, but if it occurs and is not discovered early, a lethal outcome is possible.

**Case presentation:**

We present a woman in her 80s who was known to have chronic atrial fibrillation with ischemic heart disease. She was fed through percutaneous endoscopic gastrostomy after brain injury. Erythema was observed around the stoma of the gastrostomy tube, which was later removed, and erythema extended to the left anterior abdomen. The patient was diagnosed with cellulitis. Point-of-care ultrasound examination suspected necrotizing fasciitis and, confirmed later by computerized tomography of the abdomen. The case was managed surgically.

**Discussion:**

Patients with a high clinical suspicion of necrotizing fasciitis should undergo early surgical debridement with antibiotic administration. Necrotizing fasciitis starts with a clinical picture indistinguishable from other skin infections, such as cellulitis, and imaging modalities are important for confirmation and early diagnosis. We present a case of necrotizing fasciitis after gastrostomy tube replacement for which point-of-care ultrasound played a pivotal role in confirming the diagnosis early.

**Conclusion:**

Point-of-care ultrasound is a useful adjunct tool for clinical evaluation and assessment in diagnosing early critically ill patients with life-threatening necrotizing infections.

## Highlights

• Necrotizing fasciitis is a recognized rare complication of gastrostomy tube replacement, but if it occurs and is not discovered early, a lethal outcome is possible.
• Because of scarce skin manifestations in the early stages of the necrotizing fasciitis, the diagnosis is often delayed and relies on high clinical suspicion
• POCUS is a useful adjunct tool for clinical evaluation and assessment in diagnosing early critically ill patients with life-threatening necrotizing infections.

## 1 Introduction

The incidence of peristomal infection after percutaneous endoscopic gastrostomy (PEG) placement is approximately 11 %, but necrotizing fasciitis (NF) is a rare complication with fatal outcomes [1]. It is either a polymicrobial (type 1) infection caused by aerobic and anaerobic bacteria and occurring in older patients with comorbidities and diabetes mellitus or a monobacterial (type II) invasive Group A Streptococcus infection, which may occur in any age group [2].

Early diagnosis and treatment can result in a decrease in morbidity and mortality [1]. Clinical diagnosis and high clinical suspicion are the cornerstones of diagnosing NF. Patients with a high clinical suspicion of necrotizing fasciitis should undergo early surgical debridement with antibiotic administration. In most case scenarios, NF starts with a picture indistinguishable from other skin infections, such as cellulitis, and imaging modalities are important to guide diagnosis. Point-of-care ultrasound (POCUS) may assist in early diagnosis by detecting a distinctive sonographic feature of NF and expediting management. The case report has been written in line with the 2020 SCARE Criteria [3].

## 2 Case report

A woman in her 80s was known to have chronic atrial fibrillation on anticoagulation and old non-ST-elevation myocardial infarction with a stent to the right coronary artery, hypertension, and type 2 diabetes mellitus. Previous echocardiography showed normal left ventricle size and systolic function with moderate aortic calcific stenosis. She was admitted to another facility for left atrial appendage watchman-device insertion because of a history of gastrointestinal bleeding due to duodenal AV malformation. She had cardiopulmonary arrest in that facility and ended up with hypoxic brain injury, breathing through a tracheostomy tube and feeding through percutaneous endoscopic gastrostomy (PEG). After she stayed in their care for two months, the patient was transferred to our facility for further care.

During her stay in the general medical ward, the patient developed a fever (38.1 °C) and was normotensive (128/53) with tachycardia (114 beats/min) and tachypnea (26 breaths/min). On physical exam, erythema was observed around the stoma of the PEG tube, which was later removed and extended to the left anterior abdomen. The patient was diagnosed with cellulitis and received cloxacillin. On the second day, she developed hypotension, decreased urine output, and increased BUN and creatinine and was transferred to the ICU.

Her initial laboratory results were as follows: increased lactate: 2.7 mmol/L, WBC: 21.2 × 103 per mm^3^, procalcitonin: 3.2 μg/L, hemoglobin: 13.4 g/dL, sodium: 140 mmol/dL, glucose: 14.5 mmol/L, and creatinine: 162 μmol/L.

**Fig. 1:**
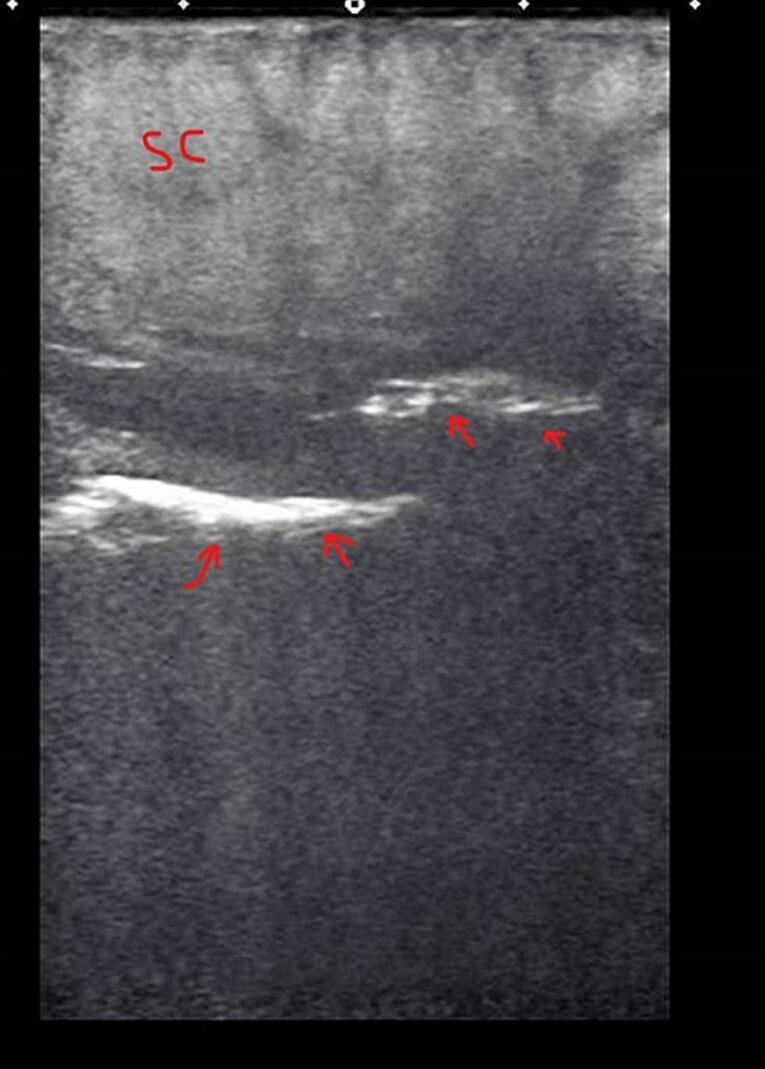
subcutaneous thickening (SC) in the anterior abdominal wall with fluid accumulation (black spaces) in the deep fascia layer, with multiple linear hyperechoic foci (arrow) representing air foci.

**Fig. 2:**
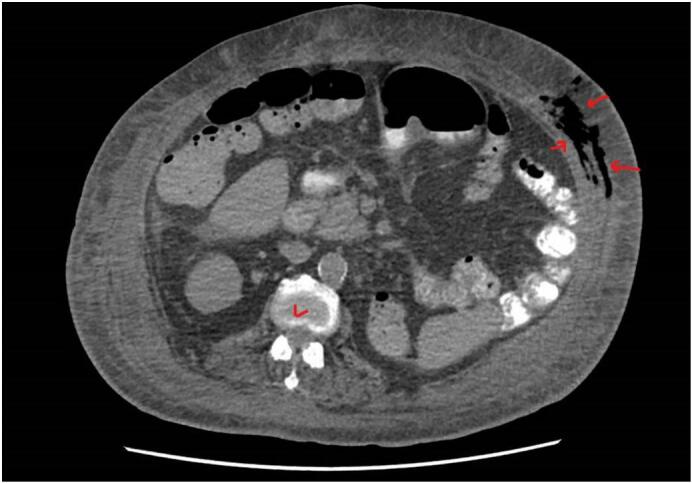
Axial CT abdomen with air in the abdominal wall (arrows); V vertebral column.

**Fig. 3:**
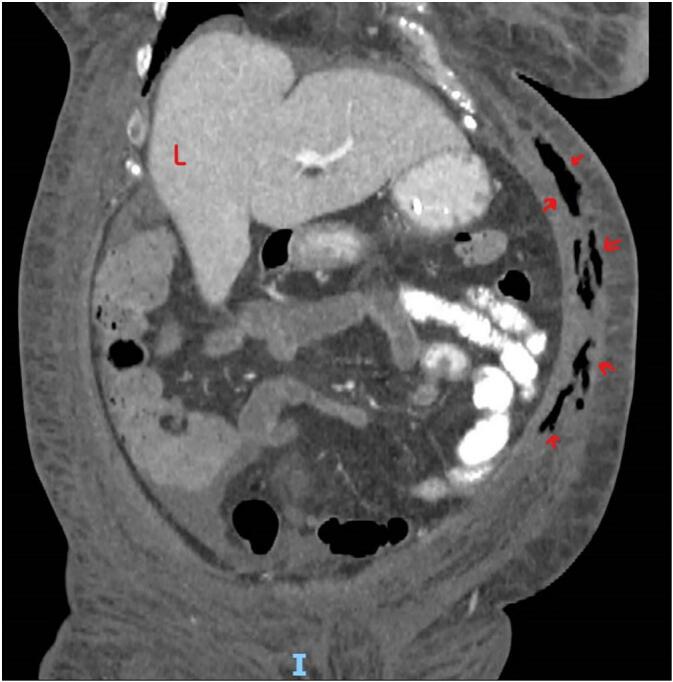
Coronal CT abdomen with air in the abdominal wall (arrows); L liver.

POCUS examination was conducted as protocol practice in our ICU when searching for source septic shock. POCUS examination of the abdomen showed subcutaneous thickening in the anterior abdominal wall with fluid accumulation in the deep fascia layer, with multiple linear hyperechoic foci seen in the left anterior abdominal wall, most likely air foci suggestive of necrotizing fasciitis (Fig. 1). Plain abdominal CT showed diffuse subcutaneous abdominal wall edema with multiple intermuscular and subcutaneous air foci in the left lateral abdominal wall (Fig. 2, Fig. 3). There was no evidence of communication with the peritoneum. Mild free fluid was seen in the right paracolic gutter and pelvis, confirming the possibility of NF (Video 1).

The following antibiotics were started empirically: meropenem: 1 g intravenously 8 h, Flagyl: 500 mg/8 h, and teicoplanin: 500 mg intravenously. She underwent operative debridement and excision of the ulcers around the PEG opening, and as the subcutaneous tissue was gangrenous, the excision was widened to include all gangrenous tissue. Curettage of all necrotic tissue overlying the anterior abdominal wall muscles was performed, resulting in bleeding from the whole surface. Hemostasis was secured, and the site was washed with H2O2 and saline, followed by packing with betadine and applying a pressure dressing.

The patient was admitted to the surgical intensive care unit postoperatively for septic shock requiring vasopressor ventilation support. She underwent repeat washouts for wound care daily for four days. Lactate levels returned to normal with a drop in the WBC count to 9 × 103 per mm3 on Postoperative Day 2. She was weaned from the ventilator and transferred to a step-down unit on Postoperative Day 5, after which the plastic surgery department was consulted to evaluate for possible skin grafts.

## 3 Discussion

Necrotizing fasciitis is a recognized rare complication of gastrostomy tube replacement, but if it occurs, a lethal outcome is possible [4]. It is a fast-progressing infection of the deep tissue that progresses to skin manifestations such as erythema and edema, followed by bullae and skin necrosis. Organisms involved in cases of patients who are elderly and have comorbidities are usually polymicrobial (type 1) aerobic/anaerobic [5]. Patients with diabetes mellitus, chronic renal failure, malignancy, and other chronic diseases that cause low immunity are at an increased risk for this fatal complication. [4] Locally, the presence of high-pressure traction can lead to necrotizing fasciitis [4]. This complication, necrotizing fasciitis, requires early, high clinical suspicion, POCUS recognition, and immediate surgical intervention with antibiotic administration.

Because of scarce skin manifestations in the early stages of the disease, the diagnosis is often delayed and relies on high clinical suspicion [6]. CT scanning helps delineate the extent of the spread of the infection and is more accurate in detecting soft-tissue gas than plain radiographs [6]. Computed tomography and MRI facilitate the detection of fascial thickening, focal fluid collection, and clinically undetectable soft-tissue gas production in anaerobic conditions and cases of diabetes, which appear necessary for the production of clinically detectable quantities of gas and retention [7,8]. Both imaging modalities have sensitivities between 80 % and 100 %; however, CT and MRI may not be readily available in an emergent setting, and risks are associated with the interdepartmental transfer of critically ill patients from the ED to the radiography department. In addition, these imaging studies require exposure to toxic contrast media in critically ill patients with compromised renal function. Early diagnosis with the help of fluorodeoxyglucose-positron emission tomography/computed (FDG-PET/CT) imaging has been reported in case reports [9]. Especially when it comes in the context of paraneoplastic diseases such as chronic lymphocytic leukemia, Hodgkin's lymphoma, and myelodysplastic syndrome and play a valuable complementary role and awareness could be lifesaving due to early optimal treatment in the disease course [9].

The ultrasonic diagnosis criteria were studied compared to histopathological pictures using a high-frequency linear probe. The ultrasonographic diagnosis of necrotizing fasciitis was based on diffuse thickening of the subcutaneous tissue and a layer of fluid accumulation more than 4 mm in depth along the deep fascia layer [10]. Ultrasonography in diagnosing NF revealed a sensitivity of 88.2 %, a specificity of 93.3 %, and a positive predictive value of 83.3 % [10]. The previously mentioned criteria (subcutaneous tissue thickening, air, abnormal fluid accumulation in the deep fascia layer) are given the “STAFF” mnemonic [10]. Ultrasound has a variable sensitivity depending on the severity of the infection and the site involved; thus, it cannot rule out the disease [11]. The POCUS helped the caring physician in the ruling-in and early recognition of this grave diagnosis based on high clinical suspicion and ultrasonic features.

Necrotizing infection is associated with significant mortality, even with optimal therapy [12]. Observational studies have reported mortality rates of 21 % to 34 % [12]. Early diagnosis and treatment are essential, with radical surgical debridement and antibiotic therapy as the primary treatment options [12]. The same was done with the present case report with a relatively early diagnosis and radical surgical treatment that necessitated skin grafting after recovering from sepsis.

The limitation of the current report is that it is a case report that needs more generalizability and represents the entire population. Because of the narrative nature, further study is needed with a control group to see the influence of POCUS in the early detection of NF (Fig. 3).

## 4 Conclusion

POCUS is a useful adjunct tool for clinical evaluation and assessment in diagnosing early critically ill patients with life-threatening necrotizing infections.

The following is the supplementary data related to this article.

Video 1 Multiple linear hyperechoic foci (arrow) represent air foci, proceeded by subcutaneous thickening. Video 1

## Consent

Written informed consent was obtained from the patient next of ken for publication of this case report and accompanying images. A copy of the written consent is available for review by the Editor-in Chief of this journal on request.

## Ethical approval

The case report was exempt from ethical approval in Ahmadi hospital, Kuwait oil company institution. The institute waived approval for all the case reports.

## Funding

No funding was obtained for this study.

## CRediT authorship contribution statement

**Zouheir Ibrahim Bitar**; manuscript preparation, writing, editing, review.

**Ossama Sajeh Maadarani**; data acquisition, and help in manuscript preparation.

**Aishah Alfarhan**; data acquisition, and help in manuscript preparation.

**Mohamed Elsayed Elhabibi**; data acquisition, and help in manuscript preparation.

**Mahmoud Mostafa Elzoueiry**; reviewing content in terms of writing principles and help in literature search.

## Guarantor

Zouheir Ibrahim Bitar.

## Research registration number

Not applicable.

## Declaration of competing interest

The authors declare no conflicts of interest.
